# Primary cutaneous *Mycobacteria avium* complex infection in a systemic lupus erythematosus patient: A case report and review

**DOI:** 10.1097/MD.0000000000041450

**Published:** 2025-02-07

**Authors:** Qi-Hao Yao, Xiu-Jiao Xia, Jun-Zhu Xu, Hong Shen, Yang Yang, Ze-Hu Liu

**Affiliations:** a Department of Dermatology, Hangzhou Third People’s Hospital, Hangzhou, Zhejiang, China; b College of Animal Science and Technology, College of Veterinary Medicine, Key Laboratory of Applied Technology on Green-Eco-Healthy Animal Husbandry of Zhejiang Province, Zhejiang Provincial Engineering Laboratory for Animal Health Inspection and Internet Technology, Zhejiang A&F University, Hangzhou, Zhejiang, China.

**Keywords:** cutaneous infection, *Mycobacterium avium* complex, next-generation sequencing, systemic lupus erythematosus

## Abstract

**Rationale:**

Nontuberculous mycobacteria infection is becoming more and more common in clinical practice, while skin and soft tissue infection is an important part. The evaluation of the immune status of patients has certain reference value for diagnosis and treatment.

**Patient concerns:**

A 48-year-old woman developed an erythematosus nodule with purulent discharge on the right hip for 4 months. She had a history of systemic lupus erythematosus for more than 20 years, in stable control with prednisone 10 mg/d, azathioprine 50 mg/12 h, and hydroxychloroquine 200 mg/12 h. There was no trauma prior to the lesion.

**Diagnoses:**

After excluding other sites involved, the patient was diagnosed as *Mycobacterium avium* primary cutaneous infection based on laboratory culture, biopsy, and sequencing techniques.

**Interventions:**

After surgical resection, a combination of oral azithromycin, rifampicin, and ethambutol hydrochloride was given.

**Outcomes:**

The lesion healed after 4 months with no relapse.

**Lessons:**

Primary cutaneous nontuberculous mycobacteria infection should raise more attention in immunocompromised and even immunocompetent populations.

## 1. Introduction

*Mycobacterium avium* complex (MAC), as a kind of nontuberculous mycobacteria (NTM), could cause human infection affecting lung, bone marrow, lymph nodes, skin, and even disseminated sites.^[1,2]^ The incidence of the infection in immunocompromised patients, such as acquired immune deficiency syndrome (AIDS) and long-term immunosuppressive therapy users, would be higher.^[3]^ The recommended treatment usually includes ethambutol, rifabutin, and other antibiotics like clarithromycin or tetracyclines once the diagnosis was decided.^[4–6]^

Systemic lupus erythematous (SLE) is a chronic autoimmune disease with unknown etiology characterized by immune abnormalities, especially the existence of antinuclear antibodies including ds-DNAs.^[7]^ In the development of SLE, deposition of immune complexes directly causes the multiple organs damage like characteristic skin lesions and nephritis.^[7]^ At present, a consensus has been reached on the combined use of glucocorticoids and immunosuppressants represented by hydroxychloroquine for the treatment of SLE, which might lead to opportunistic infections.^[8]^

Here, we report a case of a patient in stable control of SLE developed MAC infection on the hip, cured with oral rifampicin, azithromycin, and ethambutol leaving only atrophic scars.

## 2. Case report

A 48-year-old woman was admitted to our hospital, having erythematous and abscess on the right hip with slight itch and hurt for 4 months. She had a history of SLE for more than 20 years, while initially the symptoms were controlled effectively with prednisone for 40 mg/d. After that, the dosage and type of drugs were adjusted continuously, and the patient’s condition was basically stable. At present, the patient had stable control with prednisone 10 mg/d, azathioprine 50 mg/12 h, and hydroxychloroquine 200 mg/12 h. She reported no trauma or suspected contact with a source of contamination before the onset. No responsible bacteria were detected by pus culture in the local hospital. Oral cephalosporin and external application of fusidic acid cream were given as an empirical treatment receiving no effect.

**Figure 1.**
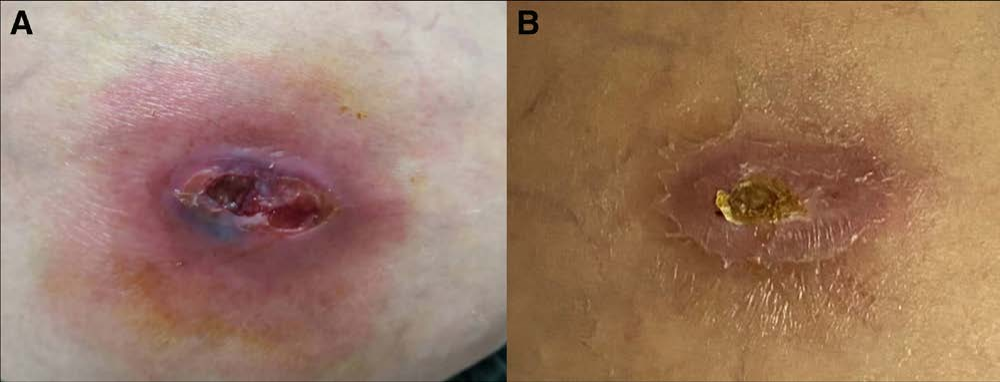
Clinical pictures of the lesion. (A) Erythematous nodule and superficial ulceration with exudate and edema, no obvious crust, pus or blisters. (B) After more than 20 d of topical application of compound polymyxin B ointment, the ulceration healed, covered with yellow scabs.

**Figure 2.**
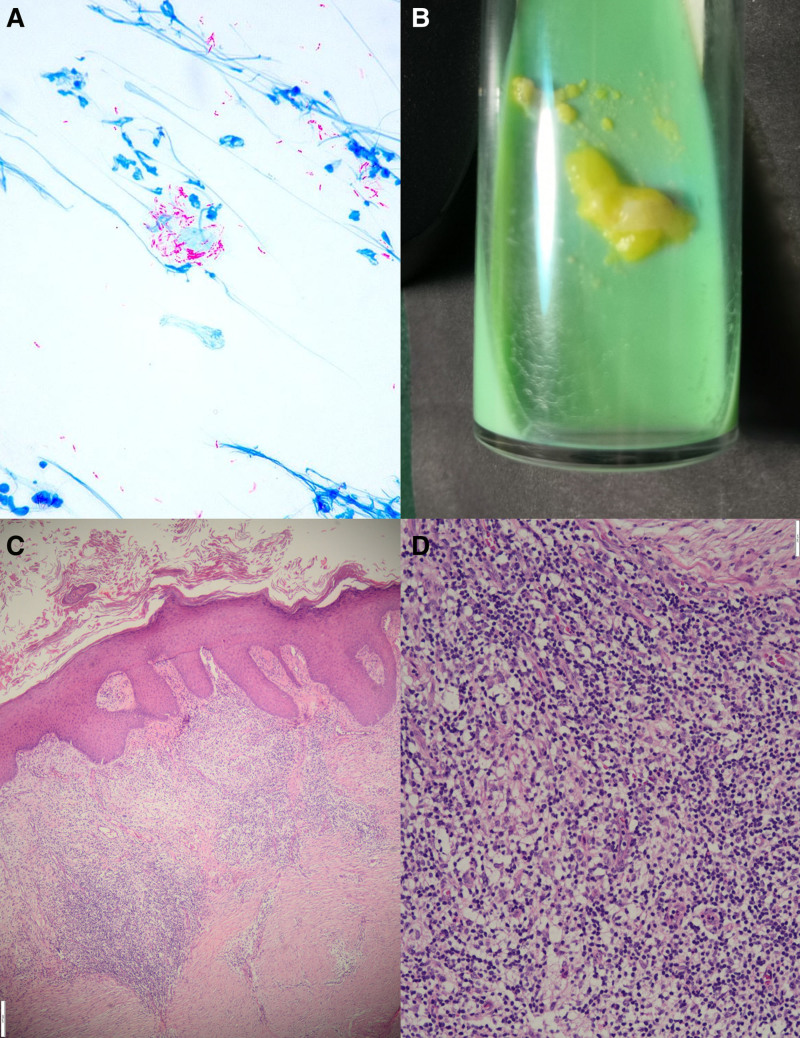
Pictures of microbiological and histopathological results. (A) Tissue smear showed multiple acid-fast bacilli (in Ziehl–Neelsen staining). (B) Yellow colonies of *Mycobacterium avium* on the modified Lowenstein–Jenson medium. (C) Hyperkeratosis, parakeratosis, neutrophil abscess in the corneum, hyperplasia and hypertrophy in the spinous layer, irregular proliferation of dermal processes (40×, in HE staining). (D) Infiltration of mixed inflammatory cells including histiocytes, lymphocytes, neutrophils, and multinucleated giant cells in the superficial dermis with dense and parallel arrangement of dermal collagen fibers around (200×, in HE staining). HE = hematoxylin-eosin.

On physical examination, no enlarged lymph nodes were touched around the body, and the heart, lungs, and abdominal organs were unremarkable. No abnormalities were found on a neurological examination. A single erythematous 4 cm × 5 cm nodule and painful superficial ulceration companied by exudate and edema was found on the right hip without elevated skin temperature, obvious pus or blisters (Fig. 1A). Direct microscopic examination of secretions is performed, showing no obvious pathogens under calcium fluorescence white staining. However, Ziehl–Neelsen stain of the smear showed acid-fast bacilli (Fig. 2A). For further diagnosis, the patient is biopsied for histopathological examination. Some tissues and tissue fluid were sent to perform metagenomic next-generation sequencing (mNGS) and culture for fungi and *mycobacteria*. While waiting for the results, compound polymyxin B ointment was empirically administered 2/d for treatment.

Histopathological examination of biopsy in hematoxylin–eosin staining showed hyperkeratosis, parakeratosis, neutrophil abscess in the corneum, hyperplasia and hypertrophy in the spinous layer, irregular proliferation of dermal processes, infiltration of mixed inflammatory cells including histiocytes, lymphocytes, neutrophils, and multinucleated giant cells in the superficial dermis with dense and parallel arrangement of dermal collagen fibers around, presenting as typical infectious granulomas (Fig. 2C and D).

Later, the patient was hospitalized for further treatment including possible debridement. At this time, the ulceration on the lesion was healed and covered with yellow scab (Fig. 1B). Most routine laboratory tests indicated no significant changes. The findings concerning SLE were as follows: antibodies to SS-A were positive, and the level of ESR, complement 3 and 4, antinuclear antibody, anti-dsDNA antibody, antibodies to RNP, CCP, Sm, SS-B, Jo-1, Scl-70, and cardiolipin all showed no meaningful results. During the hospitalization, culture on the modified Lowenstein–Jenson medium at 28°C produced yellow colonies (Fig. 2B), which was further identified as *M avium* by matrix-assisted laser desorption ionization time-of-flight mass spectrometry (MALDI-TOF). The existence of MAC was also confirmed by mNGS.

**Figure 3.**
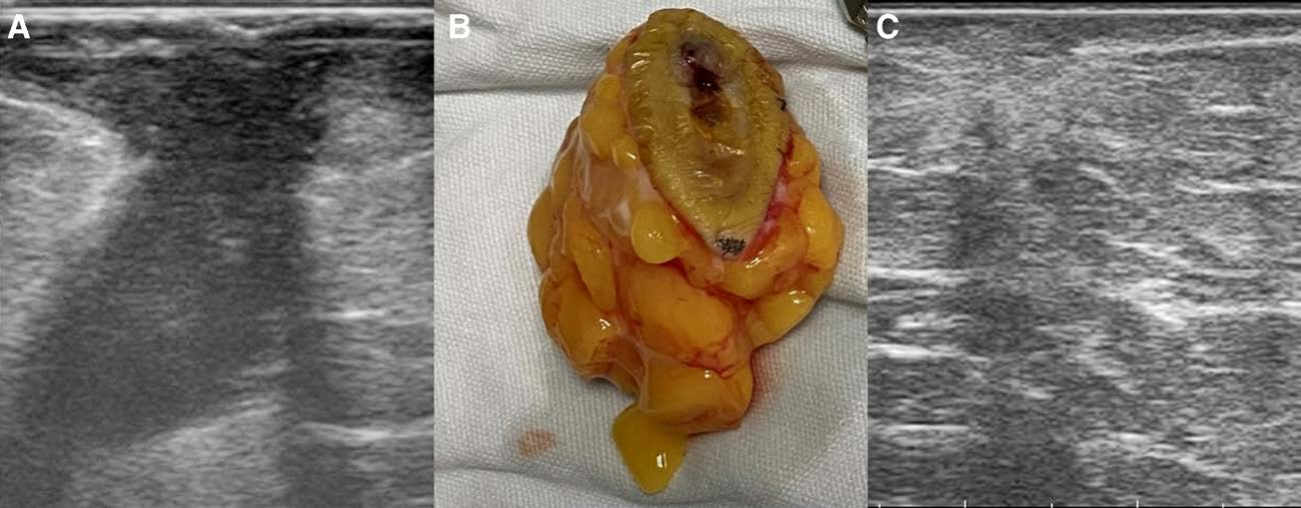
Preoperative and postoperative pictures of the granuloma. (A) B-ultrasound showed the hypoechoic structure with clear boundary under the lesion. (B) The excised granuloma and surrounding adipose connective tissue. (C) After 1 mo, the patient showed no fluid sonolucent area or new hypoechoic structure under the lesion.

Given the patient’s inactive SLE and the confirmed localized MAC infection, topical treatment was performed first. After assessing the depth of involvement using B-ultrasound (Fig. 3A), the granuloma and surrounding adipose connective tissue were surgically removed for debridement (Fig. 3B). Subsequent oral medication included a combination of oral azithromycin 0.5 g, rifampicin 0.45 g, and ethambutol hydrochloride 1 g daily. One month later, B-mode ultrasound detected no fluid sonolucent area or new hypoechoic structure (Fig. 3C). The treatment lasted for 4 months, leaving the original lesion healed with atrophic scars and pigmentation. The patient reported slight hurt and itches before a total recovery, and no similar further lesion was found in 1 year of follow-up.

## 3. Discussion

*Mycobacterium avium* complex, or also called *Mycobacterium avium-intracellulare* complex, is an important pathogen in nontuberculous mycobacteria (NTM) cutaneous infection, though the involvement was still rarely reported because of its poor pathogenicity as environmental microorganisms. Current studies suggest that this complex could be immunologically divided into more than 10 different subtypes, causing no difference in clinical manifestations.^[9]^ According to this fact, it is generally regarded as a single species complex in diagnosis and treatment.^[10]^

In addition to cutaneous involvement, MAC infection had been reported more frequently in the past decade, becoming the main driving force for the increase of NTM infection in the lung albeit for unknown reasons.^[11]^ In addition, a large number of pulmonary NTM infections were found in preexisting suspected and chronic tuberculosis patients, placing a health and economic burden on the population.^[11]^ Based on the close relationship between MAC and *Mycobacterium tuberculosis* (MTB) and the similar route of infection, the characteristics of MAC infection cases can be used as a pilot to study the mechanism of MTB infection and treatment, guiding clinical practice. In addition, though classification of MAC has limited clinical significance, sequencing technique could be used to analyze the evolutionary direction and subgroup characteristics, providing a breakthrough point for basic research.^[9]^

MAC widely exists in the environment, and the immune system has certain defense against it. At the same time, MAC itself was less virulent than MTB. Therefore, it tends to infect immunosuppressed ones, such as patients with AIDS, long-history use of immunosuppressive agents for autoimmune disease, and malignancy.^[3,12]^ In patients with inhibitory and neutralizing autoantibodies against IFN-γ (AutoAbs-IFN-γ), recurrent and refractory NTM infections including MAC infection have been reported, with the immune disturbances remaining to be elucidated.^[13]^

In addition to rare primary cutaneous infections, MAC can cause pulmonary diseases, deep infections, disseminated diseases involving multiple organs, and lymphadenitis most in children.^[1,2,10]^ In our patient, we confirmed the confined infection by evaluating the systemic imaging findings and the patient’s condition after treatment. A literature review showed that 3 out of 5 primary cutaneous MAC infection in immunocompetent people had a history of trauma.^[14]^ Two cases of lesions without trauma also occurred at exposed sites, and minor injuries to these sites not noticed by the patients may have acted as triggers. However, in our patients, the primary MAC infection occurred in the area covered by clothing and was unlikely to be related to trauma or suspicious contact to soil or fertilizers.

Examining cases presenting as cutaneous MAC infection with no disseminated manifestations or deep involvement over the last 20 years using the keywords “cutaneous infection” and “*Mycobacterium avium* complex”, we found that most patients had altered immune function, just as our patient. Table 1 showed the summarized clinical data of these patients.^[14–32]^ The infection sites were mostly located in the exposed sites such as limbs, head and face, and the skin lesions have various manifestations including nodules, ulcers, erythema, cold abscesses, with or without pus and exudate, scales, edema, vasodilatation, and paresthesia, even mimicking sporotrichoid pattern. Therefore, it may be difficult to distinguish MAC infection from other *Mycobacterium* infections morphologically.

**Table 1:** Detailed information about primary cutaneous infection of *Mycobacterium avium* complex infection.

Case	Sex	Age	Skin sites	Manifestations	Other special conditions before	Treatment
1^[15]^	M	78	Dorsal aspect of the right thumb, the first web space of the right and left hand	Several firm and crusted nodules	Invasive squamous cell carcinoma after Mohs micrographic surgery; garden hobbies for decades	Clarithromycin 500 mg bid
2^[16]^	M	49	Right forearm	A fluctuant, non-draining nodule	Dyshidrotic eczema	Not mentioned
3^[14]^	F	57	Right lower limbs	Erythematous plaques and patches with hyperkeratotic scales	No diseases, but exposure to an organic fertilizer in garden work	Minocycline 100 mg bid and hyperthermia therapy followed by clarithromycin, ethambutol, and rifampin without exact doses
5^[17]^	F	41	Left thigh	A painless ulcer	Newly-found AIDS with no treatment	Azithromycin 500 mg bid, HAART based on zidovudine, lamivudine, and nevirapine.
6^[18]^	M	63	Left chest	A reddish-brown nodule	AIDS for 8 yr without treatment for 7 yr, AIDS-associated wasting syndrome and multiple opportunistic infections	No further treatment and died
7^[19]^	F	43	Right cheek and ear	A nodular infiltrated painless plaque	None	Clarithromycin 500 mg bid, ethambutol 20 mg/kg and rifabutin 300 mg/d
8^[20]^	M	13	Right lower limb	An indurated and tender femoral node and a painful ulcer with purulent drainage	AIDS treated with HAART of zidovudine, lamivudine, efavirenz and lopinavir-ritonavir	Clarithromycin, ciprofloxacin, and ethambutol with no exact doses
9^[21]^	M	46	Right arm	A violaceous nodule	AIDS, hepatitis C, facial molluscum contagiosa, pulmonary *Mycobacterium avium intracellular*e infection, and intestinal *Clostridium difficile* infection.	Not mentioned
10^[22]^	F	7	Left breast	Tender left breast with associated erythema, induration (11 cm × 9 cm); subcutaneous seroma measuring 4 cm × 8 cm × 5.2 cm	Left breast ductal carcinoma, treated with surgery and adjuvant radiation therapy	Rifabutin 300 mg/d, clarithromycin 500 mg bid and doxycycline 100 mg bid for coexisted *Serratia marcescens*
11^[23]^	F	72	Right upper limb	An erythematous, non-fluctuant swelling involving the right forearm and back of right hand with multiple linearly arranged cystic nodules discharging pus, along the ulnar margin of the same hand	Oral methotrexate 10 mg/wk and methylprednisolone 6 mg/d for 2 yr for rheumatoid arthritis	Oral clarithromycin 500 mg/d, rifampicin 450 mg/d and moxifloxacin 400 mg/d, with surgical debridement and vacuum sealing drainage.
12^[24]^	M	56	Right foot	Painful ulcerative lesion with visible spider veins accompanied by hypesthesia and edema	Seizures, obesity, dyslipidemia, hypothyroidism, all under drug control	Oral rifampin 600 mg, clarithromycin 500 mg bid and ethambutol 1600 mg, 3/wk
13^[25]^	F	54	Right anterior thigh	Erythematous, indurated, round plaque with a central nodular component and minimal necrosis	AIDS treated with HAART for 1 mo, *Pneumocystis jiroveci* pneumonia	Oral clarithromycin, moxifloxacin, and rifabutin with no exact doses
14^[26]^	M	22	Both lower limbs	Painful erythema with subcutaneous induration	An allogeneic bone marrow transplantation for acute lymphocytic leukemia followed by a 5-yr oral prednisolone 4 mg/d	Oral clarithromycin, 400 mg/d
15^[27]^	M	42	Back and face	Erythematous infiltrated plaques and nodules with slight scales, atrophy, scars and alopecia	None	Oral regimen of clarithromycin 1 g/d, rifampicin 450 mg/d and ethambutol 750 mg/d.
16^[28]^	F	33	Right lower extremity	Redness, swelling, hardening, and a fistula with purulent discharge	Acupoint embedding therapy	Surgical debridement, with azithromycin 250 g/d, amikacin 600 mg/d, moxifloxacin 400 mg/d, and cefoxitin 4 g/d
17^[29]^	F	30	Left lower extremity	Several deep ulcers, each with a clean, granulation tissue base, surrounded by retiform, blanching erythema with admixed hyperpigmentation. Besides, there was an isolated, erythematous, blanching nodule, with no tenderness	A pedicure and leg waxing	Not mentioned
18^[30]^	M	45	Face	A crusted plaque extending from the left nasal vestibule onto the left upper lip, with bleeding sometimes	Systemic lupus erythematosus, hypertension, end-stage renal disease and depression	Not mentioned
19^[31]^	M	79	Face	A right temporal skin nodule	Primary biliary cirrhosis for 20 yr, treated by azathioprine or mycophenolate. Advanced stage and regionally metastatic cutaneous squamous cell carcinoma of the head and neck and renal cell carcinoma	Surgical excision
20^[14]^	F	59	Both lower limbs	An erythematous plaque with hyperkeratotic scale on the right shin and xerotic erythematous patches affecting the right lower leg, left shin, and left foot.	Exposure to an organic fertilizer on her legs while gardening	Minocycline hydrochloride 100 mg bid and hyperthermia therapy
21^[32]^	F	58	Left forearm	An elastic, hard nodule measuring 3.0 × 1.0 cm in size	Systemic lupus erythematosus and interstitial pneumonia for 17 yr, treated with prednisolone 17 mg/d and azathioprine 50 mg/d	Not mentioned

Abbreviations: AIDS = acquired immune deficiency syndrome, HAART = highly active antiretroviral therapy.

The most common risk factor was still AIDS, and some patients develop the disease after highly active antiretroviral therapy (HAART) to AIDS.^[20,25]^ Others include malignancy, posttransplantation and rheumatic or connective tissue diseases requiring medical treatment. The combination therapy of multiple antibiotics is widely used, and the effect is acceptable. Surgical debridement and drainage are also effective in some cases.^[23,28,31]^ In addition, we noted that primary cutaneous MAC infections with no other sites of involvement generally had better clinical outcomes in previous cases. However, in some cases not listed here, cutaneous infection can also progress to deep or disseminated ones.

In our patient, the factors affecting immunity may be SLE and its treatment. In particular, T cell’s phenotypic changes, activation abnormalities, cytokine profile transformation, epigenetic modifications and metabolic dysfunction play an important role in the pathogenesis of SLE.^[7,33]^ These could all affect the resistance to external infection. Besides, the long-term use of glucocorticoid and immunosuppressors including hydroxychloroquine, mycophenolate mofetil, cyclophosphamide, and monoclonal antibody put forward further challenges for the function of the immune system.^[34]^ We believe that both of them served as risk factors for MAC infection in our patient. In previous studies, SLE can be complicated with infections including tuberculosis, hepatitis B, pneumocystis japonicum pneumonia, etc. The control of these complications has become an important part of individualized treatment of SLE.^[35]^ In such immunocompromised populations, early prevention and screening of opportunistic infection may be more positive for patients: possible means include the conversion of glucocorticoids to more precise targeted therapy, improved vaccination, and prophylactic use of antibiotics such as SMZ-CO in high-risk groups.^[36]^

However, in clinical work the diagnosis of MAC remains a problem sometimes. In addition to distinguishing MAC from other infectious or non-infectious granulomatous diseases such as sporotrichosis, actinomycosis, and sarcoidosis, it is also necessary to distinguish MAC from other NTM, including *Mycobacterium marinum, Mycobacterium abscessus*, and *Mycobacterium chelonae*. Different NTM showed different drug sensitivity, for example, subspecies in the *Mycobacterium abscessus* complex have different responses to clarithromycin and other drugs. Therefore, it is necessary to identify the pathogen to at least a genus or even species.^[37]^ Classical methods, including Ziehl–Neelsen acid-fast stain and culture with specific media, cannot meet the need of diagnosis and treatment due to efficiency and time.^[38]^ Therefore, new molecular techniques, especially polymerase chain react-based DNA probes, mNGS and mass-spectrometric techniques, provide support for rapid identification of strains.^[16,39–41]^ In our patient, the bacterial species of the patient was once again confirmed by mNGS, which provided support for the next step of treatment.

As for treatment, combination antibiotic therapy should be preferred in patients with disseminated or focal MAC infection. Applicable drugs include clarithromycin, azithromycin and other macrolides antibiotics, tetracycline drugs, ethambutol, rifabutin, amikacin, streptomycin, and fluoroquinolones.^[4–6]^ In treatment, it is necessary to choose a combination of 2 drugs rather 1. Studies had showed that the drug resistance rate was often higher when using a single drug in MAC infections.^[42]^ In general, most patients are sensitive to macrolides plus ethambutol, and if they remain insensitive, rifampin or fluoroquinolones could be added. Active drug combination is beneficial to prevent disease progression and improve prognosis.^[43]^ For specific patients, surgical debridement may shorten the course of medication, facilitate local pus removal and skin structural reconstruction, just as in our patient. In particular, surgery may be of greater significance in MAC infections leading to bone destruction and multidrug resistance.^[44]^ In mild lesions, routine antibiotic therapy is not even required after thorough debridement.^[45]^ Therefore, antibacterial drugs and debridement combined should be emphasized in infection control, not just in MAC cases but other atypical ones.

Subsequent basic research should focus on mechanisms and treatments of MAC infection based on genetic and sequencing technologies, especially how to actively prevent MAC infection in immunocompromised people, and investigate its incidence and risk factors in normal people. Combination therapy could shorten the course of the disease, improve the prognosis, and more selectable treatment should be taken into account.

In summary, we reported a case of primary cutaneous MAC infection with SLE, treated successfully with surgical resection and combination of antibiotics. We emphasize the possibility of primary NTM cutaneous infection in immunocompromised or even immunocompetent individuals, and the need for a combination of skin biopsy, atypical bacterial cultures and sequencing technology to confirm the diagnosis.

## Author contributions

**Data curation:** Xiu-Jiao Xia.

**Funding acquisition:** Ze-Hu Liu.

**Investigation:** Xiu-Jiao Xia, Jun-Zhu Xu, Hong Shen, Yang Yang, Ze-Hu Liu.

**Methodology:** Xiu-Jiao Xia, Jun-Zhu Xu, Hong Shen, Yang Yang, Ze-Hu Liu.

**Visualization:** Qi-Hao Yao.

**Writing – original draft:** Qi-Hao Yao.

**Writing – review & editing:** Xiu-Jiao Xia, Jun-Zhu Xu, Hong Shen, Yang Yang, Ze-Hu Liu.
